# Inherited Copper Transport Disorders: Biochemical Mechanisms, Diagnosis, and Treatment

**DOI:** 10.2174/138920012799320455

**Published:** 2012-03

**Authors:** Hiroko Kodama, Chie Fujisawa, Wattanaporn Bhadhprasit

**Affiliations:** 1 Department of Pediatrics, Teikyo University School of Medicine; 2 Department of health Dietetics, Faculty of Health and Medical Sciences, Teikyo Heisei University, 2-51-4 Higashi Ikebukuro, Toshima-ku, Tokyo, 170-8445, Japan

**Keywords:** Menkes disease, Wilson’s disease, occipital horn syndrome, ATP7A, ATP7B, disulfiram, zinc, trientine.

## Abstract

Copper is an essential trace element required by all living organisms. Excess amounts of copper, however, results in cellular damage. Disruptions to normal copper homeostasis are hallmarks of three genetic disorders: Menkes disease, occipital horn syndrome, and Wilson’s disease.

Menkes disease and occipital horn syndrome are characterized by copper deficiency. Typical features of Menkes disease result from low copper-dependent enzyme activity. Standard treatment involves parenteral administration of copper-histidine. If treatment is initiated before 2 months of age, neurodegeneration can be prevented, while delayed treatment is utterly ineffective. Thus, neonatal mass screening should be implemented. Meanwhile, connective tissue disorders cannot be improved by copper-histidine treatment. Combination therapy with copper-histidine injections and oral administration of disulfiram is being investigated. Occipital horn syndrome characterized by connective tissue abnormalities is the mildest form of Menkes disease. Treatment has not been conducted for this syndrome.

Wilson’s disease is characterized by copper toxicity that typically affects the hepatic and nervous systems severely. Various other symptoms are observed as well, yet its early diagnosis is sometimes difficult. Chelating agents and zinc are effective treatments, but are inefficient in most patients with fulminant hepatic failure. In addition, some patients with neurological Wilson’s disease worsen or show poor response to chelating agents. Since early treatment is critical, a screening system for Wilson’s disease should be implemented in infants. Patients with Wilson’s disease may be at risk of developing hepatocellular carcinoma. Understanding the link between Wilson’s disease and hepatocellular carcinoma will be beneficial for disease treatment and prevention.

## I INTRODUCTION

Copper is an essential element required by cuproenzymes, including cytochrome C oxidase, lysyl oxidase, dopamine ß-hydroxylase, superoxide dismutase, tyrosinase, ascorbic acid oxidase, and ceruloplasmin. When in excess, copper’s oxidative potential can induce free radical production and result in cellular damage. In particular, adequate copper nutrition is critical during pregnancy and lactation for normal infant development [1]. Thus, tight regulation of copper homeostasis, maintained by mechanisms involving uptake, transport, storage, and excretion of copper, is required [2]. Disruptions to normal copper homeostasis are fundamental features of Menkes (kinky hair) disease (MD) [3, 4], occipital horn syndrome (OHS) [5], and Wilson’s disease (WD) [6]. Each disease is caused by the absence of or defect in two copper-transporting ATPases encoded by the *ATP7A* gene (responsible for MD and OHS) [7-11] and *ATP7B* gene (responsible for WD) [12-15]. 

ATP7A and ATP7B proteins have similar functions in cells; however, the pathology and clinical manifestations associated with MD and OHS are completely different compared to WD. MD and OHS, for example, are characterized by copper deficiency, and WD by toxicity due to excess copper. This difference relates to the particular cell type expressing ATP7A and ATP7B. ATP7A is expressed in almost all cell types except hepatocytes, whereas ATP7B is mainly expressed in hepatocytes. Diagnostic approaches are mostly established for these diseases, and treatments for MD and WD have been proposed. However, unsolved problems relating to disease diagnosis and management still exist [16-18]. Here we review genetic disorders of copper transport, and highlight clinical problems relating to their diagnosis and treatment. 

## II COPPER HOMEOSTASIS

**Fig. (1):**
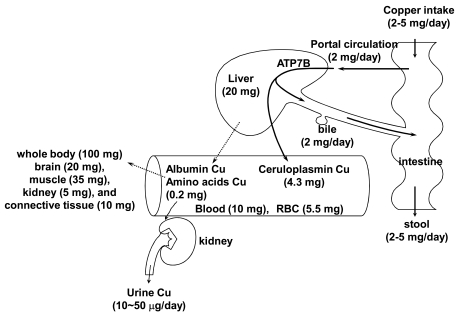
Copper metabolism in humans. ATP7B, copper-transporting P-type ATPase. Solid and dashed arrows show main and minor pathways in copper transport, respectively. Values in parentheses show amounts in adult males.

**Fig. (2):**
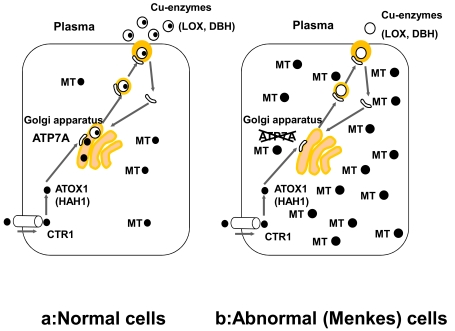
Copper metabolism in normal cells versus those affected by Menkes disease. CTR1, copper transporter 1; ATP7A, copper-transporting P-type ATPase ; ●, copper. Left, copper metabolism in normal cells. Right, copper metabolism in cells affected by Menkes disease. In cells affected by Menkes disease, copper cannot be transported from the cytosol to the Golgi apparatus. As a result, copper accumulates in the cytosol and cannot be excreted from the cells. Copper deficiency in the Golgi apparatus results in a decrease in the activities of secretory copper enzymes such as lysyl oxidase (LOX) and dopamine β-hydroxidase (DBH).

**Fig. (3):**
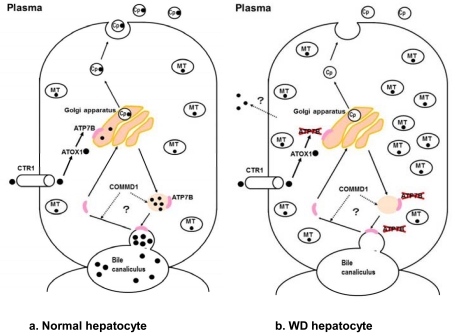
Copper metabolism in normal and abnormal (affected by Wilson’s disease) hepatocytes. CTR1, copper transporter 1; ATP7B, copper-transporting Ptype ATPase ; Cp●, ceruloplasmin; ●: copper. Left, copper metabolism in normal hepatocytes. Right, copper metabolism in hepatocyte of patient with Wilson’s disease. In hepatocytes affected by Wilson’s disease, copper cannot be transported from the cytosol to the Golgi apparatus due to a defect in ATP7B, so copper accumulates in the cytosol. Copper deficiency in the Golgi apparatus results in reduced secretion of copper into the blood as ceruloplasmin, during which biliary excretion of copper is disturbed. Accumulated copper in the hepatocyte is released into the blood as non-ceruloplasmin-bound copper, although the mechanism is unclear.

Figure **1** highlights the general mechanism of copper metabolism in humans [17,19]. The average daily copper intake is 2-5 mg in healthy adults. Copper is predominantly absorbed in the duodenum and small intestine where it is transported into the liver via the portal vein. Most of the absorbed copper is excreted in bile, but a small fraction is excreted in urine. Several parameters affect the absorption rate of dietary copper, including age, sex, food type, amount of dietary copper, and oral contraceptives. These parameters could cause the adsorption rate to vary between 12 to 71% [20]. A study using ^65^Cu isotope showed that a daily copper intake of 0.8 mg is sufficient to maintain homeostasis in adults [21]. Figs **2a** and **3a** show copper metabolism in normal cells. The high-affinity copper transporter (CTR1) is localized to the plasma membrane and mediates copper uptake. Copper uptake occurs in the intestinal brush border; however, the specific mechanism by which dietary Cu(II) is reduced to a Cu(I) ion remains unknown [20,22]. Additional copper transporters, CTR2 and divalent metal transporter 1 (DMT1), may contribute to copper uptake in the intestine, although to a lesser extent compared to CTR1 [20,22]. 

Cytosolic copper is delivered to Cu/Zn superoxide dismutase in the cytosol, Golgi apparatus, and mitochondria via the copper chaperones, CCS2, ATOX1 (HAH1), and COX 17, respectively [17,22]. In addition, cytosolic metallothionein maintains copper homeostasis in cells [23].

The liver is the central organ that maintains copper homeostasis. In hepatocytes, copper is excreted via two major pathways: bile and blood. In the excretion pathway leading to the blood, copper is delivered to the trans-Golgi network by ATOX1, and transported across by ATP7B located on the trans-Golgi membrane. Copper is transferred as a Cu(I) ion from ATOX1 to the fourth metal binding domain of ATP7B [24]. Once in the trans-Golgi network, copper is incorporated into apo-ceruloplasmin, reduced to holo-ceruloplasmin, and then excreted as ceruloplasmin into the blood. Approximately 90% of serum copper is bound to ceruloplasmin, while the remaining 10% is bound to albumin or carried as amino acid-bound copper (non-ceruloplasmin-bound copper), which is likely the form transported into various tissues. Similarly, the pathway mediating copper excretion from the liver to bile also requires ATP7B. COMM domain-containing protein 1 (COMMD1), formerly MURR1, is also involved in copper excretion to the bile. Although most intracellular copper-binding proteins, such as ATP7A and ATP7B, bind copper as Cu(I), COMMD1 has been reported to bind copper as Cu(II) [25], and may be an important component of the intracellular system for utilizing, detecting, or detoxifying Cu(II) [26]. Bedlington Terriers, for example, have a COMMD1 defect which causes copper toxicosis in the liver due to insufficient biliary copper excretion [27]. Serum copper and ceruloplasmin levels in Bedlington Terriers are not low, indicating that ATP7B function is intact. Tao *et al* showed that the carboxyl terminus of COMMD1 dimerizes (and oligomerizes) as efficiently as its full-length counterpart, and attributed a major protein-protein interaction role to this acidic residue-rich region [28]. Indeed, ATP7B and COMMD1 may cooperate to facilitate biliary copper excretion [25-29], and may thus explain why biliary copper excretion is affected in WD.

Genetic disorders involving copper metabolism are characterized by either copper deficiency or accumulation, which manifest in the form of MD, OHS, and WD (Table **1**). Recently, missense mutations in *ATP7A,* resulting in normal protein levels but defects in copper trafficking, have been identified and reported to cause X-linked distal hereditary motor neuropathy without overt signs of systemic copper deficiency [30]. 

## III GENE, STRUCTURE, AND FUNCTION OF ATP7A AND ATP7B

The *ATP7A* gene maps to chromosome Xq13.3 and encodes a protein that is 1,500 amino acids long with a molecular weight of 165 kDa [7-9]. ATP7A protein is expressed in almost all tissues except the liver. In an animal model of MD, ATP7A is expressed in astrocytes and cerebrovascular endothelial cells comprising the blood-brain barrier, as well as in neurons and choroid plexus cells, indicating that ATP7A plays a role in intracellular copper transport in these cell types [31,32]. In contrast, the *ATP7B* gene maps to chromosome 13q14.3 and encodes a protein that is 1,411 amino acids long [12-15]. The overall sequence homology between ATP7A and ATP7B is 56%, with greater homology observed in the phosphate domain (78%), transduction and phosphorylation domains (89%), and ATP-binding domain (79%). ATP7B is predominantly expressed in the liver, kidney, and placenta, and poorly expressed in the heart, brain, lung, muscle, pancreas, and intestine. The function of ATP7B in non-liver tissues remains unclear. 

**Fig. (4):**
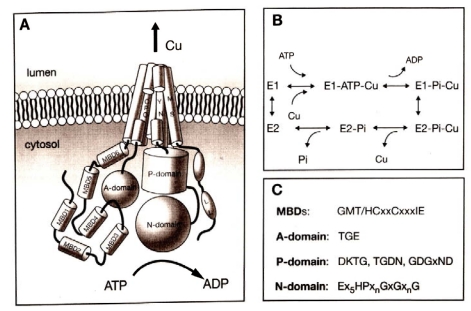
Domain organization and catalytic cycle of human copper-ATPases (ATP7A and ATP7B). **A**: membrane topology and domain organization of Cu-ATPase; MBDs, metal-binding domains; A-domain, the actuator domain; P-domain, phosphorylation domain; N-domain, nucleotide-binding domain. Modified from Lutsenko *et al.* Physiol Rev. 2007, 87: 1011. Used with permission.

ATP7A and ATP7B contain six amino-terminal metal binding domains, a phosphorylation and phosphatase domain, and eight transmembrane domains (Fig. **4**). Each protein contains six repeating motifs, GMXCXXC, that bind copper stoichiometrically as copper(I) ion at 5-6 nmol of copper/nmol of protein. This suggests that each motif binds one copper atom. ATP7A and ATP7B are predominately localized in the trans-Golgi network and transport copper from the cytosol into the Golgi apparatus. When copper levels rise inside cells, ATP7A and ATP7B traffic towards the plasma membrane to excrete excess copper [33]. Functional assays involving yeast complementation [34,35] and insect cells [36] have been reported, however, these assays are too complicated to standardize and use in a clinical test. Establishing a functional assay that can be used clinically to test for ATP7A and ATP7B activity will be beneficial not only for diagnosis of these disorders, but also to study genotype-phenotype correlations.

## IV. MENKES DISEASE (MD) AND OCCIPITAL HORN SYNDROME (OHS)

### 4.1 Genetics

Genetic disorders associated with mutations in the *ATP7A* gene are clinically divided into three categories: classical MD (referred to as MD in this review), mild MD, and OHS. MD and OHS are both X-linked recessive disorders which typically occur in male patients. In Japan, the incidence of MD is estimated to be 1/140,000 live male births [37]. Patients diagnosed with MD have a large variety of mutations in the *ATP7A* gene [17,38-40]. About 357 different mutations, including insertions and deletions (22%), non-sense (18%), missense (17%), partial deletions (17%), and splice-site mutations (16%) have been described [40]. Furthermore, genetic analysis indicates that about 75% of patient’s mothers are carriers, while the remaining 25% are not. This observation suggests that new mutations in *ATP7A* gene have been acquired in MD patients [41]. Cases of MD in females have been reported, but are rare. In a recent study, Sirleto *et al.* described 8 females who reportedly had MD, and showed that 5 of them carried X-linked chromosomal abnormalities [42].

OHS is the mildest and a rare form of MD, although the prevalence has not been reported. Mild MD has also been reported, and exhibits intermediate phenotypes between classical MD and OHS [40]. Most *ATP7A* gene mutations occurring in OHS and mild MD are splice-site or missense mutations [10,40]. Thus, residual ATP7A activity can exist [38-40]. 

Mottled mutant mice are proposed animal models of MD and OHS, and mutations in the *Atp7a* gene have been identified in these mutant mice. These brindled and macular mice show phenotypic features similar to classical MD, whereas blotchy mice have prominent connective tissue abnormalities and resemble OHS. These mice have been used in many biochemical and treatment studies [43]. 

### 4.2 Pathology 

ATP7A is localized in the trans-Golgi membrane and transports copper from the cytosol into the Golgi apparatus in almost all cell types, excluding hepatocytes. In MD, copper accumulates in the cytosol of affected cells and cannot be excreted (Fig. **2b**). Electron microscopy reveals that copper accumulates in cytoplasmic apices of absorptive epithelial and vascular endothelial cells, and in secretory granules of Paneth cells located in the intestine of macular mice [44]. Intestinal accumulation of copper results in absorption failure, which leads to copper deficiency in the body and reduced cuproenzyme activity. Copper also accumulates in cells comprising the blood-brain barrier and choroid plexus, indicating that copper is not transported from blood vessels to neurons [31,32,45,46]. The characteristic features of MD can be explained by a decrease in cuproenzyme activity (Table **2**). These enzymes include cytochrome C oxidase (localized in the mitochondria), tyrosinase, and Cu/Zn superoxide dismutase (localized in the cytosol). Decreased activity of these enzymes that are localized in the mitochondria and the cytosol in the affected cells, excluding the brain, can be improved by parenteral copper administration. 

At present, the accepted therapy involves subcutaneous copper-histidine injections. Unfortunately, cuproenzyme activity in neurons cannot be improved by treatment since copper accumulates in the mature blood-brain barrier and fails to be transported into neurons [45-47]. Neuropathological abnormalities are observed in MD, especially in the cerebral cortex and cerebellum. Brain atrophy, diffusely narrowed gyri, and widened sulci are among the abnormalities observed. Other abnormalities include loss of Purkinje cells and neuronal loss of cerebellar molecular and internal granule cell layers [48]. Neurodegeneration in MD results mainly from decreased cytochrome C oxidase activity in neurons. In addition, subdural hemorrhage occurs secondary to abnormalities in brain arteries due to decreased activities of lysyl oxidase, which causes neurological damage. 

Connective tissue abnormalities are caused by decreased lysyl oxidase activity. Lysyl oxidase combines with copper in the Golgi apparatus and is secreted from the cells. Accordingly, parenteral administration of copper-histidine cannot improve enzyme activities because the administered copper is not transported into the Golgi apparatus due to ATP7A defects. In fact, serum and urine levels of bone metabolic markers are poorly improved by copper-histidine therapy in patients with MD [49]. Neurochemical patterns in the serum and cerebrospinal fluid of patients with MD resembled that of patients with congenital deficiencies of dopamine ß-hydroxylase, suggesting that this enzyme activity is reduced in patients with MD [50]. Dopamine ß-hydroxylase is also a secretory enzyme, and thus its enzyme activity could not be increased by a copper-histidine injection. Another characteristic feature of MD is severe muscular hypotonia. Although the pathology of muscular hypotonia remains unknown, reduced activity of cytochrome C oxidase in muscles may be involved [51]. In the kidneys of macular mice, copper accumulates in the cytosol of proximal tubular cells, but not in the distal tubules or glomeruli [44] . 

In contrast, OHS is characterized by connective tissue disorders caused by decreased lysyl oxidase activity. 

### 4.3 Clinical Features 

**Fig. (5):**
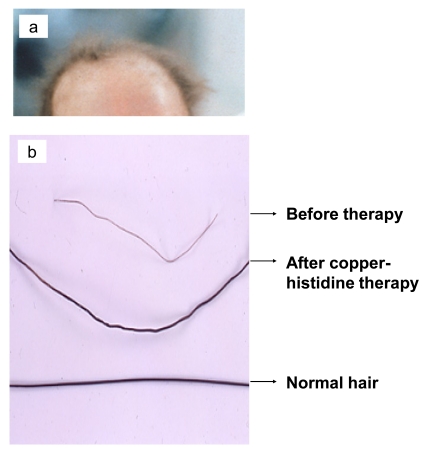
Depigmented, lusterless, and kinky hair in a 3-month-old patient with Menkes disease. Hair abnormalities were improved by copper-histidine injections.

**Fig. (6):**
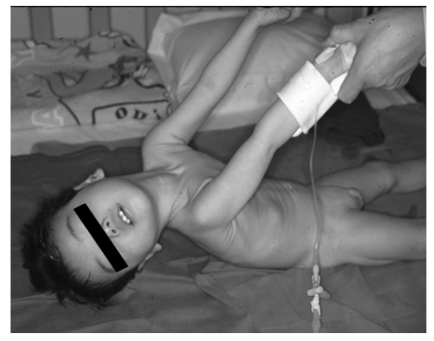
A 2 year-old patient with Menkes disease treated with copperhistidine injections since the age of 8 months. Despite treatment, he suffers from severe muscle hypotonia and cannot hold up his head.

**Fig. (7):**
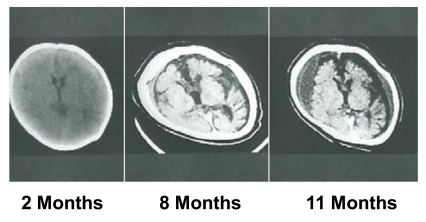
Brain CT images of a patient with Menkes disease at 2, 8, and 11 months of age. The image was taken at the age of 2 months because of a head injury. This was prior to diagnosis of MD as no neurological symptoms were observed at that time. The patient was diagnosed with MD at the age of 8 months, with brain atrophy progressing despite copper-histidine treatment. Subdural hemorrhage was observed in the patient at 11 months of age.

**Fig. (8):**
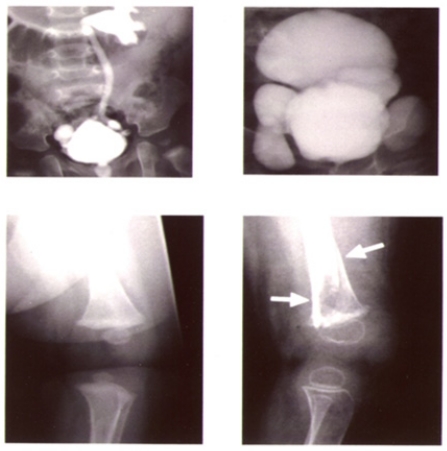
Connective tissue abnormalities in the patient with Menkes disease shown in Fig 7. Images on the left and right were taken just before treatment and at 2 years of age (also during the treatment period), respectively. Bladder diverticula formation (upper) and osteoporosis (lower) progressed despite treatment. Arrows show bone fractures.

**Fig. (9):**
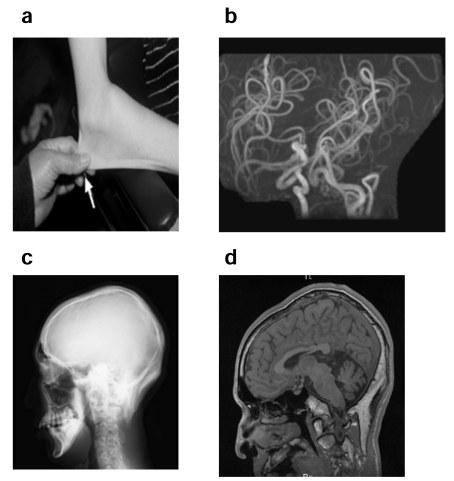
**a**) Skin laxity in a 18 year-old patient with occipital horn syndrome. **b**) MRA showing tortuosity of cerebral arteries (arrow). **c**-**d**) Occipital horns are shown in a skull X-ray (**c**) and MRI T1 WI (**d**) (arrows).

Characteristic clinical features of MD and OHS are summarized in Tables 1 and 2, and are shown in Figs. **5**-**9**. Developmental delay, seizures, and marked muscular hypotonia become prominent after two months of age when copper deficiency is advanced. Diagnosis is difficult prior to two months of age because clinical abnormalities are subtle or sometimes absent in affected newborns [52]. Neurodegeneration and connective tissue abnormalities do not improve and progress when copper-histidine therapy is initiated at 2 months of age or older. As the disease progresses, patients become bedridden and are unable to smile or speak. Although most patients die by the age of three, a few survive beyond 20 years of age [40,52].

Epilepsy, including infantile spasms, myoclonus, multifocal seizures, and tonic spasms, are observed in over 90% of patients with MD who have been treated after 2 months of age [53,54]. Magnetic resonance imaging (MRI) reveals brain atrophy and delayed myelination or demyelination, and subdural hemorrhage is often observed (Fig. **7**). Magnetic resonance angiography (MRA)reveals tortuosity of intracranial and cervical blood vessels [16]. ^1^H-magnetic resonance spectroscopy (MRS) shows a lactate peak and decreased N-acetylaspartate and creatinine/phosphocreatine levels [55]. Lesions of hypointensity on T_1_-weighted images and hyperintensity on T_2_-weighted images are transiently observed in temporal lobes, and appear similar to stroke-like lesions observed in mitochondrial myopathy, encephalopathy, lactate acidosis, and stroke-like episodes (MELAS). This suggests that the lesions observed in MD may be due to ischemic events [56]. 

Hair abnormalities, including kinky, tangled, depigmented, friable, and sparse hair, are characteristic features of MD and often diagnostic (Fig. **5**). Bladder diverticula, osteoporosis, skin and joint laxity, and arterial abnormalities are connective tissue changes caused by decreased lysyl oxidase activity. Patients with MD have intractable and chronic diarrhea that results in severe malnutrition; however, the etiology is unclear. Urinary infection is common and most likely due to bladder diverticula. Although severe copper toxicity is not typically observed, urinary ß_2_-microglobulin levels are elevated in patients, suggesting that toxicity does occur in renal proximal tubules [57].

Clinical features of OHS include mild muscle hypotonia and connective tissue abnormalities, including exostosis on occipital bones, bladder diverticula, and skin and joint laxity (Fig. **9**). However, neurological abnormalities are milder compared to classical MD, and include ataxia, dysarthria, mild hypotonia, and mild mental retardation [40]. Clinical and biochemical heterogeneity has been reported in siblings with the same missense mutation, suggesting that clinical features depend not only on genetic, but also non-genetic mechanisms [58].

### 4.4 Diagnosis

Diagnosis is not difficult once clinical features, such as intractable seizures, connective tissue abnormalities, subdural hemorrhage, and hair abnormalities, appear. However, treatment with copper-histidine once neurological symptoms appear is too late to prevent neurological disorders. Thus, early diagnosis and treatment is critical for the neurological prognosis of MD. Hair abnormalities and episodes of temporary hypothermia may be clues for an early diagnosis, as these are typically observed prior to the appearance of neurological symptoms. However, diagnosing MD before the age of 2 months is difficult because hair abnormalities and temporary hypothermia are also often observed in normal, premature babies. In contrast to serum copper and ceruloplasmin levels, which are significantly lower, copper concentrations in cultured fibroblasts from patients are significantly higher, and can help to provide a definitive diagnosis. Carrier and prenatal diagnosis can be made by mutation analysis once a mutation has been identified in the patient’s family [41].**

Male patients with muscle hypotonia and skin laxity should be suspected of OHS. Such patients can be screened by a simple brain X-ray to identify exostoses on occipital bones. Because serum copper and ceruloplasmin can range from normal to low levels in patients with OHS, diagnosis of OHS cannot be made solely on the basis of serum levels of copper and ceruloplasmin. Like MD, copper concentrations are high in cultured fibroblasts from patients, and thus are useful for diagnosing OHS [39,40]. A DNA-based diagnosis is also available for OHS [38,40].

### 4.5 Mass Screening

Copper-histidine therapy prior to neurological manifestations would be more efficient if patients with MD could be identified through neonatal mass screening. Because serum copper and ceruloplasmin are physiologically low in normal infants, measuring such parameters in patients with MD would not be a useful neonatal screening method. We recently developed a screening method to test for MD based on the ratio of homovanillic acid to vanillylmandelic acid present in urine [59]. However, although a neonatal mass screening using blood samples has been performed worldwide to test for other genetic diseases, the same system using urine samples has yet to be implemented. Our method would be easily applicable if mass screening was performed using urine samples. Kaler *et al*. reported that the ratios of dopamine to norepinephrine and dihydroxphenylacetic acid to dihydroxyphenylglycol in the plasma can help with early diagnosis of MD, and that these neurochemicals can be detected by high-throughput tandem mass spectrometry, a technique which is currently used in neonatal mass screening of other inherited diseases [60]. This test would need to be adapted for mass screening to apply it as a broad strategy with public health applications.

### 4.6 Treatments 

The current treatment strategy for MD is parenteral copper administration. Among the available copper components, copper-histidine has been reported to be the most effective [61]. Copper-histidine injection improves hair abnormalities (Fig. **5**), copper concentrations in liver, and serum levels of copper and ceruloplasmin. However, neurodegeneration progresses if copper-histidine therapy is initiated after the onset of neurological symptoms. One possible explanation is that the administered copper accumulates at the blood-brain barrier and is not transported to neurons [46]. If treatment is initiated neonatally and while the blood-brain barrier is still immature, neurodegeneration can be prevented in some patients [61-64]. A recent study conducted on 24 patients with MD showed that only 12.5% of patients treated with copper during early infancy (≤6 weeks of age) retained clinical seizures. Moreover, five patients 

with known mutations resulting in partial ATP7A function had neither clinical seizures nor electroencephalographic abnormalities [65]. These findings suggest that differences in treatment response would also depend on residual ATP7A activity. Symptoms relating to connective tissue disorders are scarcely improved by copper-histidine treatment. This is explained by the fact that the administered copper cannot be transported from the cytosol into the Golgi apparatus where it is incorporated into lysyl oxidase. Patients with MD who are diagnosed and treated early show phenotypic features of OHS. Unfortunately, no treatment trials have been reported in patients with OHS.

**Fig. (10):**
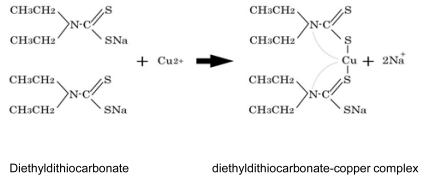
Chemical reaction of chelation by sodium N,N-diethyldithiocarbamate.

**Fig. (11):**
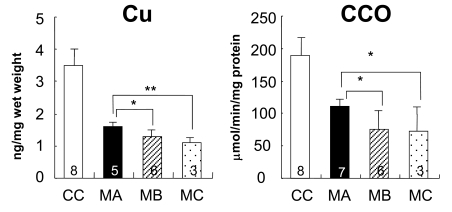
Copper concentrations (Cu) and cytochrome C oxidase (CCO) activity in the cerebrum of macular mice. MA, macular mice treated with copper and DEDTC; MB, macular mice treated with copper only; MC, macular mice without treatment. (*p<0.05; ** p<0.01.

An effective treatment for neurological and connective tissue disorders has not yet been established. If the delivery of copper into the trans-Golgi apparatus of affected cells could be achieved, then copper treatment would probably normalize the activity of lysyl oxidase and improve connective tissue disorders associated with MD and OHS. Likewise, if copper could be delivered to the Golgi apparatus within cells comprising the blood-brain barrier, copper would reach neurons and be incorporated into cuproenzymes including cytochrome C oxidase in the neurons. We previously reported that combination therapy with copper and diethyldithiocarbamate (DEDTC, Fig. **10**), a lypophilic chelator, improves copper concentration, cytochrome C oxidase activity, and catecholamine metabolism in the brains of macular mice (Fig. **11**) [66]. Takeda *et al* reported a 3-year-old patient treated with copper-histidine and oral disulfiram (DEDTC dimmer) for a period of 2 years [67]. Serum copper and ceruloplasmin levels increased and were higher than those when patients were administered copper-histidine alone. In addition, we observed a smile from the patient administered the combination therapy. The hydrophobicity of DEDTC seems to support passage of copper chelated with this compound through the membrane. To establish the utility of this therapy, further studies focusing on survival, biochemical parameters, and clinical outcome in both animal models and patients with MD are necessary. 

## V WILSON’S DISEASE (WD)

### 5.1 Genetics 

The global prevalence of WD is approximately 1/30,000 newborns, although this varies across populations [68]. This autosomal recessive disorder is caused by mutations in the *ATP7B* gene, and over 480 mutations have been reported (http//www.medgen.med. ualberta.ca/database). The R778L substitution is the most common mutation occurring in Asian patients, while the H1069Q mutation is mostly seen in European patients [17,69,70]. A correlation between genotype and phenotype has not been found in patients with WD, although several mutations correlate well with an early onset of the disease [71,72]. WD manifestations may be influenced by gene variants of baculoviral IAP repeat-containing protein 4/X-linked inhibitor of apoptosis protein (BIRC4/XIAP), which is anti-apoptotic and likely acts as a regulator of copper-induced cell death [73]. Gupta *et al* recently reported that a 9-year-old and 6-month-old patient with high neurological predominance and mild hepatic symptoms not only had heterozygous mutations in *ATP7B*, but also had mutations in *COMMD1* [74]. The authors, however, concluded from a genetic analysis of 108 patients that *COMMD1* variants do not contribute to the phenotypic heterogeneity observed in WD.

Two animal models have been reported for WD. Long-Evans Cinnamon (LEC) rats harbor a deletion in the *ATP7B* gene, accumulate large amounts of copper in the liver, and develop chronic hepatitis, which eventually leads to hepatocellular carcinoma [75,76]. Toxic milk mice have a mutation in the transmembrane domain of *atp7b*, and show decreased levels of ceruloplasmin with accumulation of copper in the liver, which eventually leads to cirrhosis [77].

### 5.2 Pathology

Copper cannot be transported from the cytosol into the Golgi apparatus, where copper is incorporated into apo-ceruloplasmin in hepatocytes of patients with WD. Accordingly, secretion of copper as holo-ceruloplasmin into the blood is affected. Excretion of copper into bile is also affected (Fig **3b**), resulting in copper accumulation in the liver. During the early stage of the disease, copper is diffusely distributed as metallothionein-copper in the hepatocytic cytosol. With disease progression, copper accumulates in the lysosomes. This excess copper induces free radical production, which causes cellular damage via oxidative stress. Furthermore, serum levels of ceruloplasmin decrease, at which point ceruloplasmin-bound copper decreases in the serum. Excess copper in the liver is released into the plasma as non-ceruloplasmin-bound copper, i.e., copper bound to albumin or amino acids, although the release mechanism is unclear [78]. The increase in serum non-ceruloplasmin-bound copper results in elevated urinary copper excretion and copper deposition in various tissues, including the brain, kidney, cornea, muscle, bone, and joint [17].

Iron as well as copper reportedly accumulates in the liver of patients with WD [79]. Due to the oxidase activity in ceruloplasmin, which converts ferrous iron to ferric iron, decreased ceruloplasmin levels in WD disrupt iron homeostasis [80]. Liver damage in patients with WD may be caused, in part, by iron accumulation, which is also toxic to the liver [79]. 

### 5.3 Clinical Features 

**Fig. (12):**
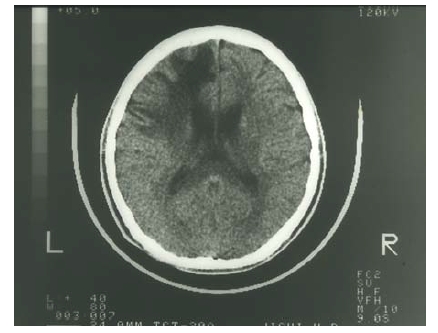
Loss of left frontal cerebral white matter in a neurological patient with Wilson’s disease who suffered right hemiplegia.

Prominent clinical features of WD include hepatic and neurological/psychiatric symptoms. Hepatic symptoms range from acute and chronic hepatitis to cirrhosis and fulminant hepatic failure. Although serum levels of transaminases are high in infants with WD, hepatic disorders usually occur after 8 years of age. Neurological symptoms appear after 12 years of age and are characterized by extrapyramidal effects, which include dysarthria, dystonia, tremor, choreoathetosis, and ataxia [81]. Cognitive impairment and depression are also common in patients with WD. Seizures occur with a prevalence of 4-8.3% and are sometimes associated with decoppering therapy [82]. The types of seizures can vary, and include generalized tonic-clonic (grand mal), simple partial, complex partial, and partial seizures with secondary and generalized periodic myoclonus [82]. Early diagnosis and initiation of treatment is crucial, especially for patients with neurological symptoms. Copper levels in the cerebrospinal fluid are elevated in patients with neurological symptoms, but decrease to normal ranges following treatment. Thus, copper levels could be a useful marker for monitoring patients with neurological symptoms [83]. “Face of giant panda sign,” tectal plate hyperintensity, central pontine myelinosis (CPM-like), and concurrent changes in basal ganglia, thalamus, and brainstem are observed in MRIs from patients with neurological WD [84,85]. High signal T_1_ images, similar to those in portal-systemic encephalopathy, are also observed [85]. In addition, loss of cerebral white matter has rarely been reported (Fig **12**). ^31^P- and ^1^H-MRS indicate that reduced breakdown and/or increased synthesis of membrane phospholipids, as well as increased neuronal damage in basal ganglia, occur in patients with neurological WD [86].

**Fig. (13):**
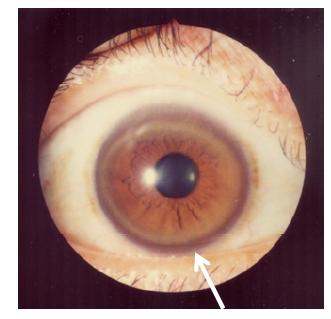
Kayser-Fleischer rings.

Initial symptoms, such as microscopic hematuria, proteinuria, hemolytic anemia, epistaxis, arthritis, cardiomyopathy, dysrhythmias, hyperpigmentation (similar to Addison’s disease), cataracts, amenorrhea, and hypersalivation, vary and make early diagnosis difficult [17,87,88]. Kayser-Fleischer rings are also common in neurological WD, which reflect copper deposition in the brain (Fig **13**). However, about 40% and 20% of patients with hepatic and neurological symptoms, respectively, show no Kayser-Fleischer rings [88-90].

### 5.4 Diagnosis

Guidelines for the diagnosis of WD were approved in 2008 by the American Association for the Study of Liver Diseases (AASLD) [89]. Diagnosis is based on low serum copper and ceruloplasmin levels (<20 mg/dL; immunoassay), high copper concentrations in the liver (>250 µg/g dry weight), high copper excretion in the urine (>100 µg/day), and by conducting a penicillamine challenge test (urinary copper excretion >1,600 or 1,057 µg/day) [89.91]. In some patients with WD, however, serum copper and ceruloplasmin levels are not low [88,89]. In fact, serum copper levels are often high in patients with WD suffering from acute liver failure due to the release of accumulated copper in hepatocytes. Furthermore, other hepatic diseases, including autoimmune hepatitis and intrahepatic cholestasis, may affect serum copper measurements and make diagnosis difficult. DNA-based diagnosis (e.g., high-resolution melting analysis or HRM) has also been reported [92]. However, approximately 17% of patients diagnosed with WD based on clinical symptoms and biochemical data have no mutations in the coding regions of *ATP7B* [69]. Scoring systems for the diagnosis of WD have been proposed in order to account for the deficiencies of any one test [93]. Although a diagnosis can be made in the vast majority of cases, a small number of patients cannot be diagnosed with the tests described above [94]. Once a patient is diagnosed with WD, all first- and second-degree relatives should also be screened for the disease. Treatment should be offered to presymptomatic patients, although diagnosis in some cases can be challenging.

### 5.5 Screening 

Clinical manifestations of WD show considerable variation, making early diagnosis challenging. The median time interval between presentation of initial symptoms and diagnosis is 18 months (ranging from 1-72 months) for patients with neurological symptoms and 6 months (ranging from 2-108 months) for patients with hepatic symptoms [90]. Despite current strategies, the mean delay from presentation of initial symptoms to diagnosis is two years (ranging from 0.08-30 years) [95]. This is mainly due to the low awareness and index of suspicion by primary care physicians [95]. Awareness and diagnosis could be improved by implementing medical education strategies that target primary care physicians.

Early diagnosis is possible through mass screening strategies, which also enable the detection of presymptomatic patients. Holo-ceruloplasmin detections in newborn blood or in urine of 3-6 year-old children have been proposed as potential mass screening strategies [96-98]. To date, however, mass screening has not yet been implemented anywhere. The specificity and sensitivity of these methods require further investigation, as well as a cost-benefit analysis when applied at the population level. Ultimately, innovative methods that allow mass screening for WD need to be developed. 

### 5.6 Therapy

The therapeutic aim for WD is to remove excess copper that accumulates in the body. When patients are diagnosed with WD, they should be promptly treated with chelating agents, including penicillamine and trientine, and/or zinc (Table **3**) [89]. Chelating agents should be taken on an empty stomach because food prevents their absorption. These agents are usually recommended to be taken 1 hour before or 2 hours after meals. The treatment choice depends on hepatic or neurological manifestations, severity of symptoms, pregnancy, and presymptomatic conditions [89,99]. In addition, patients should avoid food and water containing high concentrations of copper.

Treatment should continue throughout the patient’s life, with routine monitoring of serum and urine copper, blood cell counts, coagulation parameters, and testing for liver and renal function [100]. Kayser-Fleischer rings disappear completely in most patients who receive the full treatment [100]. Urinary copper excretion increases above 1000 µg/day for a few months following penicillamine or trientine treatment (initial treatment). These levels range between 200-500 µg/day during maintenance therapy with a chelating agent [89]. 

#### Penicillamine

While penicillamine is the most effective treatment for removing copper through urine excretion, it is associated with severe side effects [101]. These side effects include immunological conditions (e.g., lupus-like reactions, nephrotic syndrome, myasthenia gravis, and Goodpasture syndrome), skin defects (e.g., degenerative changes and elastosis perforans serpiginosa), and joint disorders (e.g., arthropathy). Given these side effects, trientine is now the preferred method of treatment [89,99]. 

#### Trientine

**Fig. (14):**
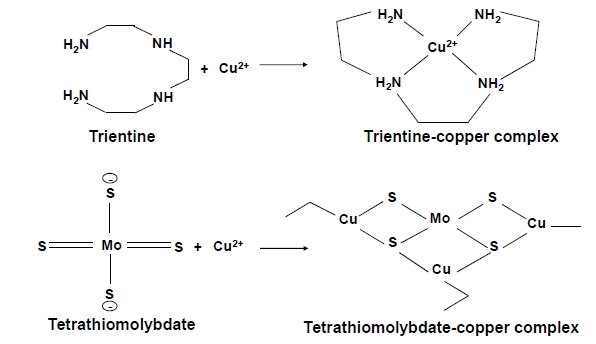
Chemical reaction of chelation by trientine (upper) and tetrathiomolybdate (lower) [126].

Figure **14** shows the chemical structure of trientine. Trientine is known to remove copper from the blood compartment, and increases urinary copper excretion. Zinc and iron are also excreted with trientine, although in lesser amounts [102]. Trientine shares some of penicillamine’s side effects, but appears to be significantly less toxic and as efficacious as penicillamine [103]. For this reason, trientine is the recommended chelator for treatment of patients with hepatic WD [99]. 

#### Zinc

Zinc is a recommended treatment for presymptomatic patients and for maintenance therapy of WD [99]. Zinc treatment of patients with WD results in increased levels of non-toxic zinc-bound metallothionein. The enterocyte metallothionein induced by zinc inhibits copper uptake from the intestinal tract, resulting in a negative copper balance [104]. Zinc is also thought to protect against copper toxicity in the liver by promoting sequestration of free copper in a non-toxic, metallothionein-bound form [105]. Treatment adequacy is determined by measuring non-ceruloplasmin-bound copper levels in the serum (5-15 µg/dL), 24-hour urinary copper excretion (<75 µg/day) [89], or by spot urinary copper excretion with less than 0.075 µg/mg creatinine [106]. Non-ceruloplasmin-bound copper levels in the serum can usually be calculated from serum copper and ceruloplasmin levels using the following equation:

non-ceruloplasmin-bound copper levels in the serum (µg/dL) = serum copper level (µg/dL) – 3 x serum ceruloplasmin level (mg/dL)

This is possible because approximately 3.15 µg of copper is bound to one mg of ceruloplasmin. 

#### Tetrathiomolybdate (TTM)

TTM is an anti-copper drug with a unique mechanism of action developed for patients with neurological WD. It has 4 sulfur groups that allow it to form a tripartite and stable interaction with copper (Fig **14**). If given with food, TTM forms a stable complex with copper, rendering it unavailable for absorption. When given without food, however, it is well absorbed and complexes with free serum copper. TTM treatment does not result in serum copper spikes typically observed with penicillamine and trientine [107]. This may explain why neurological worsening is rare with TTM treatment versus other chelating agents [108], although a patient receiving TTM treatment was reported to develop status epilepticus [109]. While TTM is now preferred over other chelating agents for treatment of neurological WD, the FDA recently decided that further studies are required before it can be used in patients with neurological WD (from HP of Pipex Parmaceuticals Comp).

#### Patients with Neurological Symptoms

In patients with neurological symptoms, clinical worsening is observed during the first few weeks of treatment in approximately 50% and 26% of patients treated with penicillamine and trientine, respectively. In addition, 25% of patients treated with penicillamine are at risk of permanent neurological damage and may not recover to baseline levels of function [110]. Neurological worsening during initiation of anti-copper therapy is attributed to spikes in levels of serum non-ceruloplasmin-bound copper which occur during mobilization of large stores of copper in the liver [107]. Although neurological worsening is also observed in a few patients treated with zinc, which is slow-acting, zinc alone or combination therapy with zinc and trientine are now recommended in patients with neurological WD [99,111,112]. Another problem is that neurological symptoms sometimes do not completely subside with treatment. Liver transplantation in some patients with neurological disorders was reported to resolve neurological symptoms associated with WD. However, detailed neurological evaluations in these patients were not carried out [113]. Because early treatment is critical in patients exhibiting neurological disorders, medical education efforts targeting primary care physicians should be implemented in order to improve early diagnosis [81].

#### Patients with Hepatic Symptoms

Patients with mild and moderate liver disorders are initially treated with chelating agents (trientine preferred over penicillamine) [89,99]. Serum levels of aminotransferases and non-ceruloplasmin-bound copper are normalized a few months after initial treatment, reaching adequate urinary copper excretion levels that range between 200-500 µg/day. Once this occurs, maintenance therapy is initiated with zinc alone or with a lower dose of chelating agents (i.e., trientine). In patients with fulminant hepatitis or hemolysis, liver transplantation is the most likely solution [114]. 

One major obstacle regarding long-term treatment of patients with WD is poor drug compliance. A recent report showed that 25% of patients were not persistently taking their medication, resulting in deterioration and occasionally fatal outcomes [115]. Accordingly, it is important for physicians to make an effort to promote compliance during therapy.

Hepatocellular carcinoma (HCC) has become an important issue for patients with WD as current treatments have improved life expectancy. In a previous study, we examined the characteristics of 25 WD patients with HCC and compared them to non-WD patients with HCC in a cohort from the Liver Cancer Study group in Japan, 1994-2003 (LCS-J) [17]. The average age at diagnosis of HCC in WD patients was considerably lower compared to non-WD patients. In addition, male to female ratios were high in WD patients. Taken together, these results show that patients with WD (mainly males) are in danger of developing HCC despite treatment. The mechanism that leads to carcinogenesis in WD remains unknown and is currently under investigation. LEC rats harboring a deletion in *ATP7B* develop HCC [76]. Tsubota *et al* reported that mRNA expression of tumorigenic proteins, Ras GTPase-activating-like protein (IQGAP1) and vimentin, was induced by persistent oxidative stress in the liver of LEC rats, making these proteins important clinical targets for HCC [116]. Production of oxygen and nitrogen reactive species, and unsaturated aldehydes that arise from copper overload in patients with WD has been reported to cause mutations in the p53 tumor suppressor gene [117]. These findings suggest that oxidative stress is associated with HCC. Vitamin E may act as an antioxidant adjunct for WD therapy [118]. The copper chelating agent, TTM, inhibits angiogenesis, fibrosis, and inflammation [119,120]. However, how these affect HCC development is unclear. Elucidation of these mechanisms will help devise strategies aimed at preventing HCC in patients with long-term WD.

## Figures and Tables

**Table 1.** Characteristics of Inherited Copper Transport Disorders in Humans

Characteristics	Menkes Disease	Occipital Horn Syndrome	Wilson’s Disease
Inheritance	X-linked recessive		Autosomal recessive
Prevalence	1/140,000 male births	Rare	1/30,000-1/35,000
Responsible gene	*ATP7A*		*ATP7B*
Gene location	Xq13.3		13q14.3
Gene product	Copper-transporting P-type ATPase (ATP7A)		Copper-transporting P-type ATPase (ATP7B)
Expression	Almost all tissues except liver		Liver, kidney, placenta, lung, brain, heart, muscle, pancreas, and intestine.
Mutations	No common mutations	Splice-site mutations, missense mutations	R778L and H1069Q substitutions are common in Asian and European patients, respectively.
Pathogenesis	Defect of intestinal Cu absorption; reduced activities of Cu-dependent enzymes	Partial defect of intestinal Cu absorption; reduced activities of Cu-dependent enzymes	Copper toxicosis; defects of biliary Cu excretion and Cu incorporation into ceruloplasmin in the liver; copper accumulates in various tissues
Clinical features	Severe neurological degeneration, abnormal hair, hypothermia, and connective tissue disorders	Connective tissue disorders, gait abnormalities, muscle hypotonia	Liver diseases, neurological diseases and psychiatric manifestations, Kayser-Fleischer rings, hematuria, arthritis, cardiomyopathy, and pancreatitis
Laboratory features	Decreased serum Cu and ceruloplasmin, and increased Cu concentrations in cultured fibroblasts	Slightly decreased serum Cu and ceruloplasmin, increased Cu concentrations in cultured fibroblasts, and exostosis on occipital bones	Decreased serum Cu and ceruloplasmin, increased urinary Cu excretion, and increased liver Cu concentration
Treatment	Cu-histidine injections		Chelating agents (e.g., penicillamine, trientine), zinc and liver transplantation
Animal models	Macular and brindled mice	Blotchy mouse	Long–Evans Cinnamon (LEC) rat Toxic milk mouse

**Table 2.** Cuproenzymes and Symptoms Due to Decreased Activity (Symptoms of Menkes Disease)

Enzyme (Localization in Cells or Characteristics)	Function	Symptoms
Cytochrome C oxidase (mitochondria)	Electron transport in mitochondrial respiratory chain, energy production	Brain damage, hypothermia, muscle hypotonia
Lysyl oxidase (secretory enzyme)	Crosslinking of collagen and elastin	Arterial abnormalities, subdural hemorrhage, bladder diverticula, skin and joint laxity, osteoporosis, bone fracture, hernias
Dopamine ß-hydroxylase (secretory enzyme)	Norepinephrin production from dopamine	Hypotension, hypothermia, diarrhea [121] *
Tyrosinase (cytosol)	Melanin formation	Hypopigmentation
Sulfhydryl oxidase (cytosol)	Keratin cross-linking	Abnormal hair
Cu/Zn superoxide dismutase (cytosol)	Oxidant defense: superoxide radical detoxication	CNS degeneration [121]
Peptidyl α-amidating monooxygenase (secretory enzyme [122])	Neuropeptide bioactivation	Brain damage
Ceruloplasmin (secretory enzyme)	Ferroxidase, Cu transport	Anemia
Hephaestin** (membrane bound enzyme [123])	Ferroxidase in enterocytes, involved in iron absorption	Anemia**
Angiogenin** (secretory enzyme [122])	Induction of blood vessel formation, antimicrobial host defense [125]	Arterial abnormalities, enteric infections [126] **
Amine oxidases**	Oxidation of primary amines, cancer growth inhibition and progression [127]	Carcinogenesis [127] **
Blood clotting factors V, VIII**	Blood coagulation system [128]	Blood clotting [128] **

* Diarrhea is often observed in patients with MD, but the relation with dopamine ß-hydroxylase is unclear.

** The relation with copper metabolism and MD is unclear.

**Table 3.** Pharmacological Therapy for Wilson’s Disease

Drug	Mode of Action	Maintenance Dose	Side effects
Trientine	Induction of urinarycopper excretion by chelating action	750-1,000 mg/day three times a day; children, 20-25 mg/kg/day	Gastritis, in rare cases aplastic anemia and sideroblastic anemia, neurological deterioration during initial phase of treatment (about 26% [130])
D-Penicillamine	Induction of urinary copper excretion by chelating action	750-1,000 mg/day three times a day; children: 20 mg/kg/day	Fever, rash, proteinuria, lupus-like reation, aplastic anemia, leukopenia, thrombocytopenia, nephrotic syndrome, degenerative change in skin, elastosis perforans serpingosa, serous retinitis, hepatotoxicity, neurological deterioration during initial phase of treatment (about 50% [110])
Zinc	Blockage of copper absorption by inducing metallothionein in enterocytes	150 mg/day, three times a day; children: 50-75 mg/day	Gastritis, biochemical pancreatitis, zinc accumulation, possible changes in immune function
Tetrathiomolybdate	Detoxifying copper in plasma and blocking copper absorption by complexation with copper	20 mg, three times with meals and three times between meals [108]	Anemia, neutropenia, hepatotoxicity, neurologic deterioration during initial treatment (about 4% [108])
